# Treatment of multiple system atrophy using intravenous immunoglobulin

**DOI:** 10.1186/1471-2377-12-131

**Published:** 2012-11-01

**Authors:** Peter Novak, Arlene Williams, Paula Ravin, Omar Zurkiya, Amir Abduljalil, Vera Novak

**Affiliations:** 1 Department of Neurology, University of Massachusetts Medical School, D55 Lake Avenue North, Worcester, MA, 01655, USA; 2 Clinical Trials Unit, University of Massachusetts Medical School, Worcester, MA, USA; 3 Department of Radiology, Harvard Medical School, Boston, MA, USA; 4 Department of Radiology, The Ohio State University, Columbus, OH, USA; 5 Division of Gerontology, Beth Israel Deaconess Medical Center, Harvard Medical School, Boston, MA, USA

## Abstract

**Background:**

Multiple system atrophy (MSA) is a progressive neurodegenerative disorder of unknown etiology, manifesting as combination of parkinsonism, cerebellar syndrome and dysautonomia. Disease-modifying therapies are unavailable. Activation of microglia and production of toxic cytokines suggest a role of neuroinflammation in MSA pathogenesis. This pilot clinical trial evaluated safety and tolerability of intravenous immunoglobulin (IVIG) in MSA.

**Methods:**

This was a single-arm interventional, single-center, open-label pilot study. Interventions included monthly infusions of the IVIG preparation Privigen®, dose 0.4 gram/kg, for 6 months. Primary outcome measures evaluated safety and secondary outcome measures evaluated preliminary efficacy of IVIG. Unified MSA Rating Scale (UMSARS) was measured monthly. Quantitative brain imaging using 3T MRI was performed before and after treatment.

**Results:**

Nine subjects were enrolled, and seven (2 women and 5 men, age range 55–64 years) completed the protocol. There were no serious adverse events. Systolic blood pressure increased during IVIG infusions (p<0.05). Two participants dropped out from the study because of a non-threatening skin rash. The UMSARS-I (activities of daily living) and USMARS-II (motor functions) improved significantly post-treatment. UMSARS-I improved in all subjects (pre-treatment 23.9 ± 6.0 vs. post-treatment 19.0±5.9 (p=0.01). UMSARS-II improved in 5 subjects, was unchanged in 1 and worsened in 1 (pre-treatment 26.1±7.5 vs. post-treatment 23.3±7.3 (p=0.025). The MR imaging results were not different comparing pre- to post-treatment.

**Conclusions:**

Treatment with IVIG appears to be safe, feasible and well tolerated and may improve functionality in MSA. A larger, placebo-controlled study is needed.

## Background

Multiple system atrophy (MSA) is a sporadic late-onset progressive neurodegenerative disorder [1]. The prevalence of MSA is 1.9 to 4.9 in 100,000 people [2,3]. MSA predominantly affects the central nervous system and results in a combination of parkinsonism, cerebellar syndrome, and dysautonomia with orthostatic hypotension. The disease progresses relatively rapidly with a mean survival of 6 to 9 years. Pharmacological management remains limited, and at present, there are no therapies that modify disease progression [4].

MSA is predominantly a white matter disease that is associated with widespread myelin degeneration and secondary neuronal loss [5]. The neuropathological hallmark of MSA is the presence of oligodendroglial cytoplasmic inclusions [6] (glial cytoplasmic inclusions) staining positively for α–synuclein [7]. Additional feature of MSA is aggregation of the filamentous α–synuclein in the neurons in several brain regions. It is believed that α–synuclein play a major role in MSA since α–synuclein aggregation occurs in the oligodendroglia and neurons in its early stages [8].

The cause of MSA remains unknown. Several lines of evidence suggest that inflammation could contribute to neurodegeneration in MSA [9-11]. Microglia are the primary immune effector cells in the brain. Activated microglia can mediate the tissue injury through secretion of toxic cytokines, complement proteins, and free radicals that can lead to the degeneration of myelin, axonal dysfunction, and neuronal death. Activation of microglia [9-11] and upregulation of several inflammatory genes [12] have been described in patients with MSA.

Intravenous immunoglobulin (IVIG) has anti-inflammatory properties with multiple mechanisms of action. IVIG inhibits autoreactive T cells, suppresses autoantibodies through anti-idiotypic interactions and interferes with the production of cytokines [13]. IVIG is effective in the treatment of several autoimmune or neuroinflammatory disorders.

This pilot clinical trial was based on the hypothesis that the neuroinflammatory activity in MSA can be altered by using IVIG. Preliminary results were presented in abstract form [14].

## Methods

### Participants

This was a single-arm, interventional-, single-center, open-label prospective study. Patients with a history of probable MSA [15] were enrolled in the study (Table 1). All patients had some combination of cerebellar syndrome and parkinsonism, poorly responding to levodopa; autonomic failure, wherein systolic blood pressure dropped ≥ 30 mm Hg within 3 minutes of standing, and urinary incontinence. Cerebellar findings included at least one of the following: ataxic gait, cerebellar dysarthria, cerebellar oculomotor findings or limb ataxia. To minimize the chance of enrollment of non-MSA patients, the presence of all three syndromes (autonomic, parkinsonism, and cerebellar) was required even though, in the consensus criteria for probable MSA, concurrent occurrence of both parkinsonism and cerebellar syndrome is not required for diagnosis [15,16]. Brain MRI was performed in all subjects to rule out structural abnormalities that can mimic MSA. Additional exclusion criteria included the presence of dementia and volume depletion.

**Table 1:** Characteristics of subjects


Age in years (median, range)	59, 55-64
Gender (women/men)	2/5
Disease duration (median, range)	5, 2-14
MSA Type (cerebellar/parkinsonian)	5/2
Levodopa (number of patients)	3
Amantadine (number of patients)	3
Proamatine (number of patients)	3
Fludrocortisone (number of patients)	2
Pyridostigmine (number of patients)	2

The severity and progression of the disease was evaluated by the Unified MSA Rating Scale (UMSARS), a validated and disease-specific instrument [17]. UMSARS part I (UMSARS-I) evaluates activities of daily living, part II (UMSARS-II) evaluates motor functions, part III (UMSARS-III) evaluates autonomic functions, and part IV (UMSARS-IV) evaluates disability. Blood pressure measurements of UMSARS-III were obtained automatically with the use of Dinamap ProCare Monitor 100 (GE, Fairfield, CT).

### Imaging

Anatomical images were acquired on a 3T GE HDX MRI scanner using three-dimensional magnetization prepared rapid gradient echo (MP-RAGE) images to quantify volumes of white and gray matter in the anatomical regions of interest. MP-RAGE images of individual participant were co-registered, and then registered to the standard anatomical template using the Statistical Parametric Mapping software package (SPM, Wellcome Department of Imaging Neuroscience, University College, London, UK) [18,19]. MR findings were compared to 37 age and gender matched healthy controls that participated in the same imaging protocol. We calculated volumes in the frontal, parietal, temporal, occipital, and cerebellar regions and their respective subregions. Details of segmentation and data processing were published previously [20].

### Interventions

We used Privigen® [21], a commercial preparation that is a high-purity 10% liquid IVIG stabilized by L-prolin. Privigen® retains the Fc and Fab functions of the immunoglobulin G (IgG) molecule since heating and the chemical or enzymatic treatment of the preparation are avoided. The median half-life of Privigen® is 36.6 days.

Interventions included monthly infusions of Privigen® with the dose being equal to 0.4 gram of the preparation per kilogram of body weight, for 6 months. This dose is recommended for treatment of primary humoral immunodeficiency [21,22]. The dose remained unchanged on gram per kg basis but did change proportionally with body weight changes during the study. Premedication was not routinely administered. Premedication including acetaminophen, antihistamines, and oral prednisone was permitted only patients experienced an infusion related adverse event (AE).

Outcome measures: The primary outcome measure was to evaluate the safety and tolerability of the IVIG infusions in patients with MSA. The primary endpoint was defined as the frequency of AEs. AEs including their severity and relationship to the IVIG were assessed throughout the study and at least 60 days after the last infusion. The AEs were considered to be related to the IVIG infusion (infusional AE) if they occurred during an infusion or within 72 hours afterwards. Non-infusional AEs were further classified as possible related to IVIG or likely not related to IVIG. The safety and tolerability end points using IVIG are unknown for MSA. For the primary immunodeficiency diseases, the FDA recommends the cutoff limit 40% for all AEs [23].

The secondary outcome measure was to evaluate the preliminary efficacy of IVIG for the treatment of MSA. The primary efficacy endpoint was change of the UMSARS ratings compared to baseline. The secondary efficacy end points were changes of quantitative volumetric imaging also compared to a baseline.

### Protocol

The experimental protocol included a screening visit, 6 infusion visits a month apart, and a final visit. Creatinine and blood urea nitrogen were obtained at each visit to assess kidney function and hydration status. The levels of IgA were also obtained at the screening to rule out IgA deficiency since Privigen® contains IgA in trace amounts. CRP was obtained at the screening and final visit. The UMSARS ratings were obtained at each visit before infusions. Imaging was done on the same day or within few days after the screening visit and within one month after last infusion. The approved protocol for IVIG infusions at the University of Massachusetts Medical School was based on manufacturer’s recommendations [21]. For each infusion adverse events were documented during the infusion and by follow up interview 3 days later.

Subject received IVIG by infusion pump. Infusions began at the rate of 0.5 mg/kg/min for 15 minutes in sitting or semi recumbent position. All subjects were closely supervised. The subject’s vital signs (heart rate, respiratory frequency, blood pressure, and temperature) were measured before the infusion, than twice in 15-minute intervals, then again every 30 minutes until the end of the infusion. The infusion rate was gradually increased each time vital signs were measured until the maximum rate of 4 mg/kg/min was achieved. If significant changes in vital signs occurred the infusion rates were usually slowed down or kept the same until the vital signs were again stable.

#### Statistical analysis

One-way ANOVA for repeated measures test was used to compare the difference between analyzed variables obtained at baseline, during treatment, and after treatment. All statistical analysis was done using JMP 8.0 (SAS Institute, Inc., NC).

#### Standard protocol approvals, registrations, and patient consents

The study was approved by the Institutional Review Board of the University of Massachusetts Medical School and the Institutional Review Board of the Beth Israel Deaconess Medical Center, where MR imaging was performed. All subjects signed informed consent forms.

## Results

Twelve subjects were screened, three subjects failed to meet the inclusion criteria, nine participants were enrolled in the study, and seven completed the protocol. Demographic characteristics and medications are in the Table 1. The disease duration was calculated from the onset of the first symptoms, which proceeded the time of diagnosis. IgA deficiency was ruled out in all subjects.

### Medications

There have been some changes in medications in spite of efforts not to change any medication for the trial duration. Subject #4 discontinued proamatine and fludrocortizone because of subjective improvement. Subject #3 discontinued levodopa because of questionable efficacy. The neurologist treating subject # 2 increased the dose of ropinirole because of worsening of parkinsonism.

#### Adverse Events

There were 42 IVIG infusions. No serious AEs were observed and most of subjects tolerated the treatment protocol well.

### Safety

Table 2 summarizes the frequency of non-serious adverse symptoms observed for all infusions. There were 43 infusional AEs. However, excluding BP-related infusional AEs, there were 11 infusional AEs (frequency of occurrence 26.2%). There were additional 18 AEs that occurred beyond 72 hour limit (frequency of occurrence 42.8%). The most common infusional AE was an immediate and transient elevation of the systemic blood pressure (BP). 100% subjects (occurrence 33 times out of 42 infusions) experienced an increase of systolic BP ≥ 20 and diastolic BP ≥10 mg Hg at least once. The BP elevation usually occurred within minutes of the infusion onset, necessitating a reduction in the infusion rate, changes of position more upright, interruption of infusion and/or allowing the patient to void. ANOVA showed a significant increase in systolic BP (p=0.05, mean±sd systolic BP 129.1±32.4 mmHg before infusions, 159.0±25.8 mm Hg highest systolic BP during infusions), but the increase in diastolic BP was not significant (p=0.20, diastolic BP 79.1±16.5 mmHg before infusions, 88.6±13.8 mm Hg highest diastolic BP during infusions). Skin rash was the second most common AE. Two subjects withdrew from the study because they experienced a skin rash during or immediately (minutes) after IVIG infusions.

**Table 2:** Characterizations of adverse events

	**Type**	**No**	**Subjects**	**R**	**Comments**
1	Elevated BP	7	7	I	Transient, responding to adjusting the infusion rate
2	Accidental injury	1	1	N	After It4, nose fracture
3	UTI	1	1	N	After It5, treated with antibiotics
4	Pruritic skin rash	4	3	I	2 subjects withdrew from the study. In these subjects, one subject experienced rash after It1 and second after It2. 1 subject experienced rash after I4 and I5 and was treated with **Diphenhydramine**, **Acetaminophen** and oral Prednisone
5	Increased temperature and/or skin flushing	5	1	I	Treated with **Diphenhydramine**, **Acetaminophen**, and slowing the infusion rate
6	RLS	1	1	N	Diagnosed after It4, treated with iron supplements
8	Decreased GFR	1	2	N	After It5 and It6, resolved
9	Ankle edema	1		N	After It5, treated with diazide diuretics
10	Elevated BUN	1	1	N	After It5, started trial with elevated BUN at screening visit, resolved
11	Worsening of allergies	2	1	N	Worsening of running nose and cough after It5 and It6, also associated with cold and fever after It6, treated with **Acetaminophen**, **Pseudoephedrine**, and **Diphenhydramine**.
13	Nodular lung abnormality	1	1	N	Later determined to be abnormal tangle of veins, probably since birth
14	Low potassium	1	1	P	After It1, treated with increased dose of potassium
15	GI viral infection	1	1	N	After It5, treated with fluids, antiemetics and anti-diarrheal agents
16	Elevated PSA	1	1	N	Before It1, resolved
17	Wrist strain	1	1	N	After It1, resolved
18	Worsening of sleep apnea	1	1	N	After It2, prrescribed CPAP

Legends: UTI=urinary tract infection, BUN=blood urea nitrogen, GFR=glomerular filtration rate, PSA=prostate specific antigen, CPAP=continuous positive airway pressure. R= relationship of the AE to the study drug, N= not related, PR=possibly related, I=infusional AE. It1-6 designates the infusion treatment 1–6.

### Functional measure

**Figure 1:**
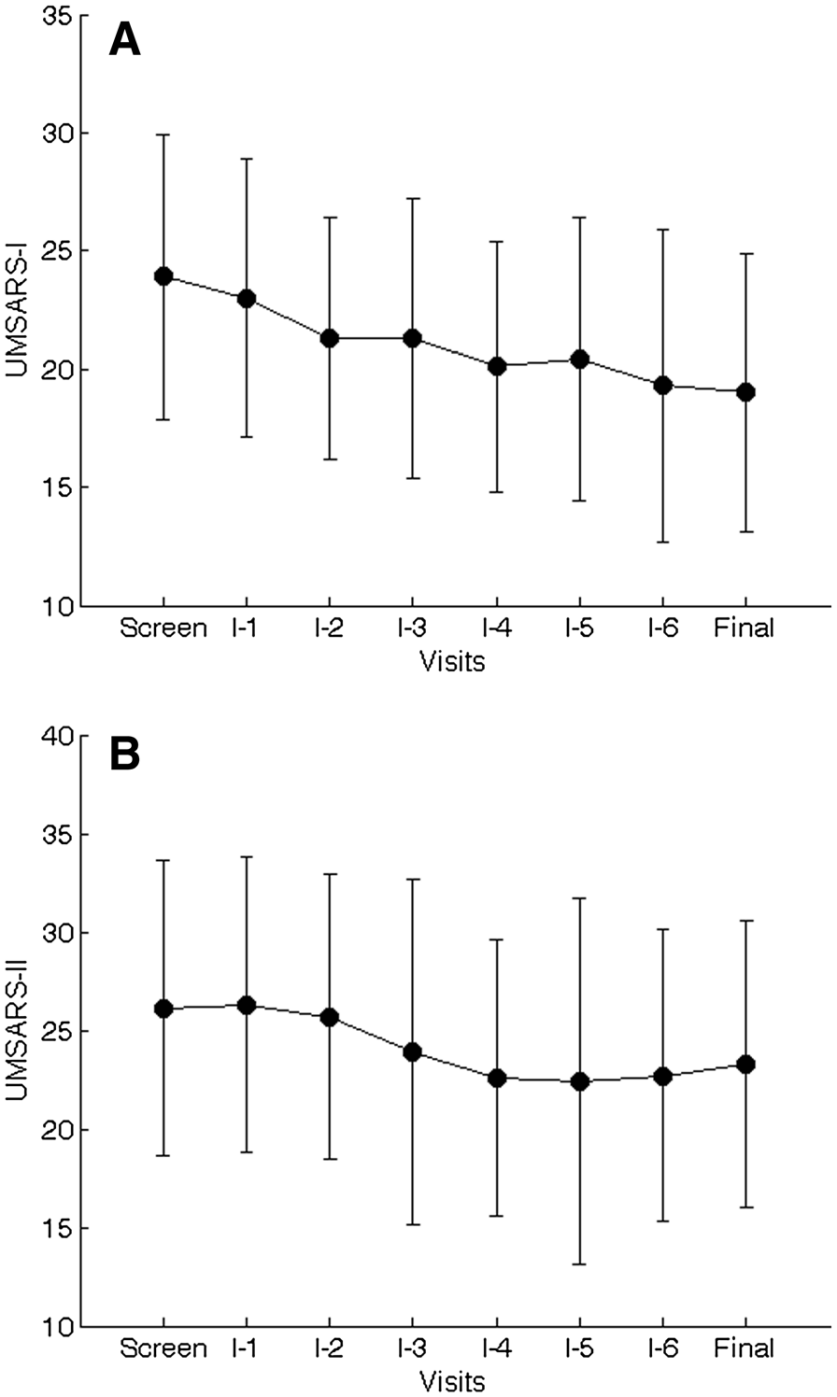
**Average UMSARS scores.** Each dot represents an average score at each visit. The bars represent standard deviations. **A**=UMSARS part I scores, **B**=UMSARS part II scores.

UMSARS-I was improved in all 7 subjects (Figure 1) (p<0.01). UMSARS-II improved in 5 subjects (p<0.025), was unchanged in 1 subject and was worse by 1 point in 1 subject who had developed a severe cold a few days before the final visit. Comparison of the final-visit and the baseline-visit ratings shows a significant decrease in UMSARS-I (p=0.0128) and UMSARS-II (p=0.025). The differences in UMSARS-III (systolic BP p=0.67, diastolic BP p=0.45) and UMSARS-IV (p=0.36) were not significant. C-reactive protein levels did not change with treatment (p=0.25).

Imaging did not show differences in gray and white matter volumes in post treatment MSA subjects. As compared to controls, MSA subjects had lower gray matter volume (p<0.05) in cerebellum, putamen and hippocampus bilaterally. White matter volume was increased (p<0.05) in the frontal lobe bilaterally, right parietal lobe, right superior temporal gyrus, left middle temporal gurus, cerebellum and putamen bilaterally. There were no significant differences in volumes compared before and after interventions.

## Discussion

This pilot open label study investigated the safety and preliminary efficacy of using IVIG over a 6-month period for treatment of MSA. IVIG treatment was well tolerated. Post-treatment functional assessment showed promising improvement of many areas of daily living activities. IVIG infusions were acutely associated with increase in blood pressure, but post-treatment orthostatic hypotension did not change significantly. Imaging studies have shown brain and cerebellar atrophy.

No serious AEs occurred as a result of the total 42 IVIG infusions. Previously reported AEs associated with IVIG, such as renal failure and thromboembolic events, did not occur in our cohort. Minor AEs are common with IVIG treatment and range from 2% to 25% per infusion [24,25]. The most commonly reported AE’-associated with Privigen® is headache, which occurs in 65% of patients [21]. No headaches were observed in our group. Skin reaction occurs rarely with IVIG. In our study two subjects developed pruritic rash and they withdrew for safety reasons. It is likely that rash in one subject was not related to IVIG because it persisted beyond 3 months after the last infusion, and then it was reassociated with a food allergy. The most common AE in our study was elevated BP, which occurred in all of the subjects. This appears to be an MSA-specific effect of IVIG or Privigen® since, to our knowledge, this is the first study reporting transient elevation of the BP due Privigen® infusions. Previous studies utilizing Privigen® observed infusion induced hypotensive reactions [24-26]. However a transient BP elevation was reported with other IVIG preparations. In our cohort, elevated BP declined when the infusion rate was slowed down, or position changed from supine to semi recumbent or sitting or both. Allowing a subject to void was another effective maneuver to reduce BP.

The mechanisms leading to IVIG-induced BP elevation are unclear. BP changes were typically observed minutes after starting infusions rendering the effect of volume expansion unlikely. It also is unlikely that sensitization to IVIG plays a role in BP elevation because BP elevation occurred during the first infusion in six of seven participants. Possible mechanisms include the effect of a yet-to-be-determined substance in IVIG or a specific substance in Privigen®. Possible candidates are cytokines, vasoactive substances or other proteins, or L-prolin that is unique to Privigen®. MSA patients can have denervation hypersensitivity that can render them more sensitive to vasoactive substances in Privigen®. It is of interest to elucidate Privigen®-induced mechanisms of BP elevation given that, in general, treatment of hypotension in MSA can be difficult.

CRP as a marker of systemic inflammation did not change significantly following IVIG treatment. The main reason why the expected reduction of CRP was not observed in our trial was the fact that two subjects experienced viral infection at the final visit that increased CRP dramatically. Without these 2 subjects, the mean CRP would be reduced at the end of the trial compared to baseline.

Because a biomarker of MSA is not available, the UMSARS was chosen as a proxy for the disease stage. At present, UMSARS is the best instrument for evaluation of disease severity and disease progression [16]. Baseline UMSARS scores in our study are similar to those of a progressive observational study by the North American MSA Group of 67 patients [27]. In that study the average increases of the UMSARS score for part I and part II were equal to 3.1 and 4.5 points over 12 months. However, the European MSA study group [28] showed a faster progression of MSA as indicated by the increase in UMSARS-I by 6.7 points and UMSARS-II by 9.6 points over 12 months. In our study, group averages showed decreases in the part I and part II scores. These results are encouraging, given that current therapies for MSA are only symptomatic.

This pilot trial was open label and therefore a placebo effect cannot be ruled out. Investigators who evaluated the UMSARS (PN, PR) were not blinded to the intervention, and so the rating could be biased. Only a larger placebo-controlled double-blinded study can effectively delineate the role of IVIG in the treatment of MSA. However, Privigen® appears to have a profound immediate effect on BP, as discussed above. This fact can complicate the blinding of future studies since BP responses to infusions can distinguish the interventions from placebo. On the other hand, there was no significant difference in systemic BP on UMSARS-III based on a comparison of screening rating and the final visit rating suggesting that hypertensive effect of Privigen® is transient and therefore theoretically should not “mask” the putative disease-modifying effect. This fact is important to consider since what appears to be a slowing of disease progression could be due to symptomatic drug affect.

The effect of IVIG on MSA remains to be clarified. The present study design was based on the assumption that brain inflammation contributes to MSA. Furthermore, IVIG enters the central nervous system only if the blood brain barrier (BBB) is disrupted [29]. Although measurement of the extravasation of IVIG was never performed in MSA, BBB is impaired in MSA [30,31] including at basal ganglia and at similar areas that show activation of microglia [32]. However, it is not clear whether the disruption of blood brain barrier is necessary for IVIG to be effective with MSA. Alternatively, IVIG could exhibit its effect on systemic modulation of the immune system.

The imaging portion of the study showed significant differences in particular brain areas of MSA subjects as compared with controls. Our results are similar to those of previous studies [33-35]. Our MSA patients had reduced gray matter volume mainly in cerebellum and putamen and increased in hippocampus. White matter volume was increased in frontal lobe, putamen, cerebellum and hippocampus. The significance of the increase of the white matter remains unclear. It may simply reflect that proportionally more gray matter was lost than true enlargement of the white matter.

We also compared volumes before and after the treatment 8 months apart. There was no significant difference in any of the analyzed variables. Previous studies [33-35] showed interval progression in atrophy of brain tissue. For example, annualized rates of atrophy of MSA, parkinsonian variant, are 1.0% for the whole brain (controls 0.4%) but can be as high as 4.5% in the pons (controls 0.2%) or 3.2% in cerebellum (controls 0.3%). In our study, the volumes did not change between the pre- and post-treatment evaluations. However, our imaging comparison interval was shorter (8 months versus 1 year). These findings are also encouraging but need to be validated in larger studies with longer follow up.

## Conclusion

Treatment with IVIG appears to be feasible and well tolerated. However a larger, placebo-controlled study is needed to further evaluate a benefit to risk ratio with the use of IVIG in treatment of MSA.

## Competing interests

Peter Novak received research support from NIH 1R43NS064640 and Teva Pharmaceutical Industries. Vera Novak received research support from,1R01AG028076-A2 and 1R21- DK084463-01. Paula Raven received research support from Teva Pharmaceutical Industries, University of Massachusetts and ACADIA Pharmaceuticals.

## Authors' contributions

Trial design: PN. Data acquisition: PN, AW, PR, VN. MRI processing: AA, OZ. Data analysis and interpretation: all authors read and approved the final manuscript.

## Pre-publication history

The pre-publication history for this paper can be accessed here:

http://www.biomedcentral.com/1471-2377/12/131/prepub

## References

[B1] Wenning GK Colosimo C Geser CF Poewe W Multiple system atrophy Lancet Neurol 2004 3 93 103 10.1016/S1474-4422(03)00662-8 14747001

[B2] Schrag A Ben-Shlomo Y Quinn N Prevalence of progressive supranuclear palsy and multiple system atrophy: a cross-sectional study Lancet 1999 354 1771 1775 10.1016/S0140-6736(99)04137-9 10577638

[B3] Bower JH Maraganore DM McDonnell SK Rocca WA Incidence of progressive supranuclear palsy and multiple system atrophy in Olmsted County, Minnesota, 1976 to 1990 Neurology 1997 49 1284 1288 10.1212/WNL.49.5.1284 9371909

[B4] Wenning GK Stefanova N Recent developments in multiple system atrophy J Neurol 2009 256 1791 1808 10.1007/s00415-009-5173-8 19471850

[B5] Lantos PL The definition of multiple system atrophy: a review of recent developments J Neuropathol Exp Neurol 1998 57 1099 1111 10.1097/00005072-199812000-00001 9862632

[B6] Papp MI Kahn JE Lantos PL Glial cytoplasmic inclusions in the CNS of patients with multiple system atrophy (striatonigral degeneration, olivopontocerebellar atrophy and Shy–Drager syndrome) J Neurol Sci 1989 94 79 100 10.1016/0022-510X(89)90219-0 2559165

[B7] Wakabayashi K Takahashi H Japanese Society of Neuropathology Symposium The Frontier of Spinocerebellar Degeneration Cellular pathology in multiple system atrophy Neuropathology 2006 26 338 345 10.1111/j.1440-1789.2006.00713.x 16961071

[B8] Yoshida M Multiple system atrophy: α–synuclein and neuronal degeneration Neuropathology 2007 27 484 493 10.1111/j.1440-1789.2007.00841.x 18018485

[B9] Ishizawa K Komori T Sasaki S Arai N Mizutani T Hirose T Microglial activation parallels system degeneration in multiple system atrophy J Neuropathol Exp Neurol 2004 63 43 52 14748560 10.1093/jnen/63.1.43

[B10] Stefanova N Reindl M Neumann M Kahle PJ Poewe W Wenning GK Microglial activation mediates neurodegeneration related to oligodendroglial alpha-synucleinopathy: implications for multiple system atrophy Mov Disord 2007 22 2196 2203 10.1002/mds.21671 17853477

[B11] Banati RB Myers R Kreutzberg GW PK (‘peripheral benzodiazepine’)–binding sites in the CNS indicate early and discrete brain lesions: microautoradiographic detection of [3H]PK11195 binding to activated microglia J Neurocytol 1997 26 77 82 10.1023/A:1018567510105 9181482

[B12] Langerveld AJ Mihalko D DeLong C Walburn J Ide CF Gene expression changes in postmortem tissue from the rostral pons of multiple system atrophy patients Mov Disord 2007 22 766 777 10.1002/mds.21259 17290454

[B13] Aktas O Zipp F Regulation of self-reactive T cells by human immunoglobulins-implications for multiple sclerosis therapy Curr Pharm Des 2003 9 245 256 10.2174/1381612033392152 12570829

[B14] Novak P Ravin P White BM Williams A Novak V Treatment of Multiple System Atrophy Using Intravenous Immunoglobulins – Update Clin Autonom Res 2009 19 316

[B15] Gilman S Low PA Quinn N Albanese A Ben-Shlomo Y Fowler CJ Kaufmann H Klockgether T Lang AE Lantos PL Litvan I Mathias CJ Oliver E Robertson D Schatz I Wenning GK Consensus statement on the diagnosis of multiple system atrophy J Neurol Sci 1999 163 94 98 10.1016/S0022-510X(98)00304-9 10223419

[B16] Gilman S Wenning GK Low PA Brooks DJ Mathias CJ Trojanowski JQ Wood NW Colosimo C Durr A Fowler CJ Kaufmann H Klockgether T Lees A Poewe W Quinn N Revesz T Robertson D Sandroni P Seppi K Vidailhet M Second consensus statement on the diagnosis of multiple system atrophy Neurology 2008 71 670 676 10.1212/01.wnl.0000324625.00404.15 18725592 PMC2676993

[B17] Wenning GK Tison F Seppi K Sampaio C Diem A Yekhlef F Ghorayeb I Ory F Galitzky M Scaravilli T Bozi M Colosimo C Gilman S Shults CW Quinn NP Rascol O Poewe W Multiple System Atrophy Study Group Development and validation of the Unified Multiple System Atrophy Rating Scale (UMSARS) Mov Disord 2004 19 1391 1402 10.1002/mds.20255 15452868

[B18] Wells WM 3rd Viola P Atsumi H Nakajima S Kikinis R Multi-modal volume registration by maximization of mutual information Med Image Anal 1996 1 35 51 10.1016/S1361-8415(01)80004-9 9873920

[B19] Van Leemput K Maes F Vandermeulen D Suetens P Automated model-based tissue classification of MR images of the brain IEEE Trans Med Imaging 1999 18 897 908 10.1109/42.811270 10628949

[B20] Novak V Zhao P Manor B Sejdic E Alsop D Abduljalil A Roberson PK Munshi M Novak P Adhesion molecules, altered vasoreactivity and brain atrophy in type 2 diabetes Diabetes Care 2011 11 2438 2441 21926285 10.2337/dc11-0969 PMC3198286

[B21] Privigen® http://www.privigen.com/pdf/Privigen_PI.pdf.

[B22] Dalakas M Role of IVIg in autoimmune, neuroinflammatory and neurodegenerative disorders of the central nervous system: present and future prospects J Neurol 2006 253 Suppl 5 V25 V32 16998751 10.1007/s00415-006-5004-0

[B23] FDA Guidance for Industry: Safety, Efficacy, and Pharmacokinetic Studies to Support Marketing of Immune Globulin Intravenous (Human) as Replacement Therapy for Primary Humoral Immunodeficiency http://www.fda.gov/BiologicsBloodVaccines/GuidanceComplianceRegulatoryInformation/Guidances/Blood/ucm072130.htm.

[B24] Nydegger UE Sturzenegger M Adverse effects of intravenous immunoglobulins Drug Saf 1999 21 171 185 10.2165/00002018-199921030-00003 10487396

[B25] Pierce LR Jain N Risks associated with the use of intravenous immunoglobulin Transfusion Med Rev 2003 17 241 251 10.1016/S0887-7963(03)00038-5 14571392

[B26] Murphy E Martin S Patterson JV Developing practice guidelines for the administration of intravenous immunoglobulin J Infus Nurs 2005 28 1 8 10.1097/00129804-200507000-00009 16106210

[B27] May S Gilman S Sowell BB Thomas RG Stern MB Colcher A Tanner CM Huang N Novak P Reich SG Jankovic J Ondo WG Low PA Sandroni P Lipp A Marshall FJ Wooten F Shults CW North American Multiple System Atrophy Study Group Potential outcome measures and trial design issues for multiple system atrophy Mov Disord 2007 22 2371 2377 10.1002/mds.21734 17914727

[B28] Geser F Wenning GK Seppi K Stampfer-Kountchev M Scherfler C Sawires M Frick C Ndayisaba JP Ulmer H Pellecchia MT Barone P Kim HT Hooker J Quinn NP Cardozo A Tolosa E Abele M Klockgether T Østergaard K Dupont E Schimke N Eggert KM Oertel W Djaldetti R Poewe W European MSA Study Group Progression of multiple system atrophy (MSA): a prospective natural history study by the European MSA Study Group (EMSASG) Mov Disord 2006 21 179 186 10.1002/mds.20678 16161136

[B29] Jorgensen SH Storm N Jensen PE Laursen H Sorensen PS IVIG enters the central nervous system during treatment of experimental autoimmune encephalomyelitis and is localised to inflammatory lesions Exp Brain Res 2007 178 462 469 10.1007/s00221-006-0752-8 17091295

[B30] Bartels AL Willemsen AT Kortekaas R de Jong BM de Vries R de Klerk O van Oostrom JC Portman A Leenders KL Decreased blood–brain barrier P-glycoprotein function in the progression of Parkinson's disease, PSP and MSA J Neural Transm 2008 1157 1001 1009 18265929 10.1007/s00702-008-0030-y PMC2468317

[B31] Song SK Lee SK Lee JJ Lee JE Choi HS Sohn YH Lee PH Blood–brain barrier impairment is functionally correlated with clinical severity in patients of multiple system atrophy Neurobiol Aging 2010 32 2183 2189 20149484 10.1016/j.neurobiolaging.2009.12.017

[B32] Gerhard A Banati RB Goerres GB Cagnin A Myers R Gunn RN Turkheimer F Good CD Mathias CJ Quinn N Schwarz J Brooks DJ [11C](R)-PK11195 PET imaging of microglial activation in multiple system atrophy Neurology 2003 61 686 689 10.1212/01.WNL.0000078192.95645.E6 12963764

[B33] Brenneis C Egger K Scherfler C Seppi K Schocke M Poewe W Wenning GK Progression of brain atrophy in multiple system atrophy. A longitudinal VBM study J Neurol 2007 254 191 196 10.1007/s00415-006-0325-6 17334661

[B34] Paviour DC Price SL Jahanshahi M Lees AJ Fox NC Longitudinal MRI in progressive supranuclear palsy and multiple system atrophy: rates and regions of atrophy Brain 2006 129 Pt 4 1040 1049 16455792 10.1093/brain/awl021

[B35] Köllensperger M Wenning GK Assessing disease progression with MRI in atypical parkinsonian disorders Mov Disord 2009 24 Suppl 2 S699 S702 19877233 10.1002/mds.22582

